# Rachel Challice's Vow

**Published:** 1888-03-17

**Authors:** Lina Nevill

**Affiliations:** Author of "A Future on Trust," "Juno," &c.


### Rachel Challice's Vow.

By Lina Nevill, Author of "A Future on Trust," "Juno," &c.

### Chapter XIV.—Found.

"You may stretch your hands out towards me ;
Ah ! you will—I know not when ;
I shall nurse my love, and keep it
Faithfully for you till then."—*Adelaide Procter.*

Rachel had no difficulty in finding the house Martha had spoken of as David's home.

Every street and byeway in Bedd-gelert was familiar to her. There had been but little building since her departure, and the long straggling street which constitutes the main portion of the village is not intricate in its situation.

Her knock was answered by a fretful, feeble voice from within, telling her to "Come in."

She knew the voice in a moment, although it was sharper and less musical than formerly in its key.

Esther was sitting by the fire, with a baby of about six months in her lap, while another child, about two years old, stood holding on to her apron, ready to set up a cry of fright at the appearance of a stranger.

The room was dirty and untidy. The grate looked as though it had not been cleaned for a month, and the floor was not in a much better state. Ashes were scattered about the hearth ; a dirty, stained white cloth was on the table, the remains of the last meal still on it ; and the food itself could scarcely be called appetizing, so slovenly were all the appointments.

Then Esther herself——

Could this worn, faded-looking woman, with pinched, sharpened features, be the bright careless girl she had left behind her, a newly-made bride, not four years ago?

Where were the round, delicately-tinted cheeks, the red full-lipped mouth, the child-like look in the blue eyes? Then the former Esther had thought much of her clothes, her hair had been always dressed in the newest fashion, and she had been fond of putting on all the cheap showy jewellery she possessed. This new Esther's hair was rough, and twisted loosely into an untidy coil. She wore an old ragged dress, faded and stained, and no ornament but her wedding-ring.

Time, which had dealt so kindly with Rachel, had been very hard upon poor Esther. It had robbed her of all her fresh prettiness, while it had added real beauty to the face of her elder sister. There was no question which looked the youngest. Esther seemed almost middle-aged now beside Rachel.

"Who is that?" asked Esther, in her whining voice, not taking the trouble to look round. "Do leave me alone, Essie," she continued, giving the elder child a rough push, as, frightened at seeing a stranger, the little girl pressed closely to her mother. "What a trouble you are! Why don't you go and play with your dolly?"

The child, frightened, cross, and sleepy, set up a piteous wail, which woke the baby on Esther's lap, and cries of the two children echoed through the house.

"Esther," said Rachel gently, "it is I, dear. I have come to see you."

Esther jumped up hastily, put the crying baby into its cradle, and ran towards her sister.

"Oh Rachel, Rachel!" she sobbed. "Have you come back at last? I have wanted you so badly, and I thought you were never coming."

Rachel's heart was touched, and she pressed her sister closely to her. Yet, for all its warmth, Esther's was but a selfish welcome. There was no thought but for herself—none for Rachel. No inquiry for her—only a sort of reproach that she had kept aloof for so long, and neglected to be at hand to help her sister.

Rachel was too glad to see Esther ; too pleased that she, in her turn, was glad to see her, to notice the selfishness. And even if she had done so, it would not have hurt her. It was too much Esther's nature for Rachel to be/surprised that she thought first of herself. The wonder would have been if she had not done so.

"Have you missed me, dear? Did you want me?" she said lovingly. "I never thought of that, or I would have come before. I fancied you would be so happy with your husband that you would have forgotten your poor old sister, and would never care to see her again."

"But I did want you, Rachel," reiterated Esther. "David does not help me as you and Granny used to ; he is very hard and cross. He expects so much from me, and scolds and finds fault if it is not done. And the children take up so much of my time, and he won't let me have a girl in to look after them ; he says we can't afford it. I thought when I married that I should be well off ; but I am poorer and have to work harder than ever I did in the old days. And yet David is always grumbling, and saying I am so extravagant."

The torrent of complaints poured forth volubly. It had never been Esther's way to suffer silently.

"Poor Esther!" was all Rachel could think of to say.

Then she turned towards the children, who had ceased to cry. Essie, with wide open blue eyes, stared at the stranger, her thumb in her rosy mouth.

She was a pretty child, or would have been had she been cleaner. As it was, her face was smeared with tears and bread-and treacle ; her pinafore suffered from the same complaints, with the addition of a good deal of black dirt ; her curly light hair was rough and uncombed ; and yet she had much of her mother's former picturesque prettiness, with delicate colouring and small childish features.

"This is your eldest one? She is called after you, then. It is late for her to be up, is it not? And baby, what is her name?"

"Rachel. Yes, I suppose Essie ought to be in bed. But I was so tired, and David kept me up so long with him, I was sitting down to rest a bit when you came in. Essie, come here directly and let me undress you ; and be a good girl and don't fidget while I do it, or you shall have no treacle on your bread to-morrow."

"Come to me, Essie, and let Aunt Rachel undress you. She has a penny somewhere for a good little girl!" said Rachel persuasively, holding out her hand—an invitation and bribe which Essie, with many doubtful looks, at length accepted.

"So you called your baby after me? That was nice of you, Esther, dear," she continued.

"David wished it. I wanted Essie to be called Alice Maud, and baby Gladys ; but David laughed at me, and said such names were foolish ; no one we knew had them. I thought them very pretty and nice-sounding, much grander than Esther and Rachel ; I am tired of both of those."

"How is David, dear? I heard he was ill. I hope it is nothing much."

"Oh, no ; it is only a cold. But he is very cross and fidgety this evening ; he has given me no rest. I do hope he will soon be better ; it is dreadful getting in no money, and it was so stupid of him never to have joined a club. As for his father, he is the most dreadfully cruel old man that ever lived. He actually turned us out of doors the very day we were married."

"So I heard. I went there first, taking it for granted you lived there."

"You went to Aberelwyn, and saw old Mr. Stephens! Well, what did he say to you? Did he turn you out?"

Rachel laughed.

"Oh, no ; he was tolerably civil in his way, which is not saying much. But I am very sorry you and he are not friends. Is there no way of bringing him round?"

"Oh, no." And Esther tossed her head. "He was angry with David for marrying me. Just fancy that! As if I wasn't every bit as good as he was—nasty old fellow!"

Esther's careless words stung Rachel, and made her wish she had never persuaded David to marry her sister.

"David is so thoughtless," pursued Esther, returning to the story of her grievances. "He actually wished to see the doctor this evening, and he was here only yesterday. I told him I had no one to send, and of course I could not go myself."

"I will go up and see him presently. I am a nurse, you know ; next best to a doctor."

"A nurse!" repeated Esther vaguely. "Why did you become a nurse? You were never very fond of children. Have you a nice place, and good wages?"

"Not that kind of nurse, dear," replied Rachel, smiling at Esther's mistake. "I have been trained in a London hospital, and go out nursing sick people."

"How funny! What put the idea into your head? I never heard of anyone doing it before. Well, it suits you ;

I never saw you looking so well. You are quite young and pretty, and you never used to be much to look at; not like me," remarked Esther jealously.

"No, indeed!" assented Rachel heartily. "I never could hold a candle to you in looks. But what have you been doing to yourself, Esther? I cannot say you look as blooming as usual. Have you been ill or worried?"

"Yes; I am always ill, always worried," sobbed Esther. "I know I have grown old and ugly, and lost all my good looks. David does not care how I am dressed; he is only always telling me to be careful, and be sure not to spend too much money on my own and the children's clothes. Oh, Rachel! you don't know how dreadful it is to be married! What horrid wear and tear it is, from morning to night, looking after one's husband and children, and no thanks either in the end!"

Poor Esther began to sob piteously, swaying her weak body backwards and forwards. Little Essie, seeing her mother's tears, relapsed into a howl, while baby began sympathetically to scream in her cradle.

The noise was deafening. Rachel caught up the crying baby, and began to walk about the room, hushing it in her arms. Esther's sobs gradually subsided, and she told Essie sharply to "leave off making that noise!"

"David says the children cry so," she said, fretfully. "But I'm sure I don't know how to prevent it. I hate hearing them just as much as he does, only he never thinks of me. Everything is my fault now. Do you remember, Rachel, in the old days how different things were? Then I was always right and you wrong; Granny always used to say so. Poor dear Granny, I wish she were alive now! She would take my part against David, and tell him how cruel he is to me."

"Oh, but you would not like that, Esther!" exclaimed Rachel quickly. "You would not like anyone to interfere between you and your husband."

"I would, indeed! I think David is very cruel and unkind, and treats me very badly. I should be only too pleased if someone would tell him so. You might do it, Rachel."

For the first time in her life Rachel felt really angry with her sister. This incessant abuse of her husband by Esther disgusted Rachel, and made her for once think her blindly-loved sister in the wrong.

"No one should interfere between a husband and wife," she answered coldly. "I should be the last to do so. Now, Esther, where does this little maid sleep? She can hardly keep her eyes open."

"I will carry her upstairs," said Esther sulkily. To be found fault with at any time annoyed her; but by Rachel, her despised sister, who formerly had been the family scapegoat—such taking to task and turning of tables was too much for her equanimity.

"Esther!" called a voice from upstairs—a voice whose sound brought a rush of colour to Rachel's cheeks, and made her heart beat faster.

"Esther, come to me, quick!"

Esther uttered an impatient exclamation, caught up Essie in her arms, and began slowly to ascend the stairs.

Rachel sat quietly by the fireside, the sleeping baby, her little namesake, in her arms. She could hear Esther's step moving about upstairs, and distinguish David's voice and her's, though not what they were saying.

Presently Esther came down, with tears running down her cheeks.

"Oh, Rachel! I do think he is really bad; ever so much worse than I thought. He is in dreadful pain, he says. We must get the doctor, and I don't know who to send. I feel I should drop by the way if I tried to go."

"Is there no one who could take the message—no boy who would go?"

"Yes, Tommy Harris would, directly, if I gave him sixpence; he used to do all my messages for me."

"Then send him off at once," said Rachel impatiently. "Why did you not think of that before?"

"But I can't!" moaned Esther. "I haven't got sixpence in the house, and I owe money all over the village, as it is. Oh, Rachel! we are so dreadfully poor! I dare not tell David how deeply we are in debt; and since he has been ill there has been nothing coming in, and twice as much going out. You don't know the misery I have suffered. Fancy seeing one's husband die for want of sixpence!"

"Give that to the boy, and tell him to run all the way," said Rachel, drawing out her purse and putting a shilling in Esther's hand. "And come to me, Esther, for any money you may want. I have plenty for the present."

She turned towards the stairs as she spoke, and went up to David's room.

The door was open.

If Esther was but the wreck of her old self, what havoc had been going on here!

His wide open eyes caught sight of Rachel, and he stopped moaning when he saw her.

"Oh, Rachel! I'm real glad you've come! Has Esther sent for the doctor? Tell her to make haste. I think I am dying."

Rachel's quick, practised eyes saw the danger also. She knew what to do, and lost no time in setting about it.

"How clever you are, Rachel," he said once. "You have done me so much good, and it all seems to come naturally to you. Now, my poor little Esther can't do much but stand by and cry."

"It is my work, David. I should know no better than Esther if I had not been taught."

He was quieter before the doctor came. Rachel's remedies had eased his pain for the time.

There was grave danger. The doctor made no secret that he considered David's condition critical, and told Esther plainly that her husband's recovery was more than doubtful.

"He must not be left alone," was his order. Then looking at Esther's pale, tearful face and weak, trembling figure, he added: "Cannot you get a neighbour in to help you? You don't look fit for nursing him yourself."

"I will stay, sir," said Rachel, coming forward. "I am her sister, and a trained nurse."

"That will be better than anything. He will be safe with you," he replied. Then, giving Rachel the necessary instructions, he rode off, promising to come again early next morning.

Rachel was determined to stay, but knew she must see Mrs. Endsleigh first and ask her permission.

David had fallen into a restless slumber, which Rachel thought might last some time. Esther could sit with him while she ran up to the hotel. It would not take her more than half-an-hour altogether, and then she could relieve Esther for the night.

She hurriedly explained the state of the case to her sister, and Esther, entreating her to be quick and not leave her alone long, allowed her to go.

Nell was gone to bed; but Mrs. Endsleigh was in the sitting-room when Rachel knocked and came in.

"Has anything happened?" asked Mrs. Endsleigh, as Nurse Challice stood before her with bright, excited eyes, flushed cheeks, hair blown about by the wind, and breath coming short and quick from her rapid run to the hotel.

"I came to ask if I might stay out all night," panted Rachel. Then, seeing Mrs. Endsleigh's surprised look, she continued: "I ought to have told you before. My only sister lives in Bedd-gelert, and I fear her husband is dying. No, it is nothing infectious," she said quickly, as Mrs. Endsleigh involuntarily drew back a step. "May I stay with him to-night? My sister is not fit to sit up. I will leave my address, and I could come to Miss Endsleigh in ten minutes if she really wanted me."

"Of course we can spare you," said Mrs. Endsleigh, kindly. "Nell seemed quite well this evening—better, rather, than usual. Only, why did not you tell us before about your sister? Is that why you did not wish to come to Bedd-gelert? I cannot quite understand it all."

"Yes," said Rachel in a low voice, "that was the reason. I am very sorry; I ought to have explained at once. But we had not met for more than three years, and I foolishly fancied she would not care about seeing me again."

Almost fearing what might have happened during her absence, and what she might find awaiting her, Rachel hurried back to the cottage, and went straight upstairs.

David was still in the same restless sleep in which she had left him, while Esther had not dared to move since she began her watch. Rachel softly put down her basket, and sat down in the vacant chair by the bed-side.

For an hour she sat motionless, afraid to stir, for fear of waking David. About eleven o'clock he opened his eyes, and looked round uneasily.

His look fell full on Rachel, and the restless expression left his face.

"You are still here, Rachel?" he whispered. "Don't go. I like to have you near me."

"I will not go till you are better," she replied, though her heart sank as she spoke the words.

She knew, only too well, what "better" meant for David Stephens.

*(To be continued.)*

